# Pathologic TDP‐43 downregulates myelin gene expression in the monkey brain

**DOI:** 10.1111/bpa.13277

**Published:** 2024-05-23

**Authors:** Longhong Zhu, Dazhang Bai, Xiang Wang, Kaili Ou, Bang Li, Qingqing Jia, Zhiqiang Tan, Jiahui Liang, Dajian He, Sen Yan, Lu Wang, Shihua Li, Xiao‐Jiang Li, Peng Yin

**Affiliations:** ^1^ Guangdong Key Laboratory of Non‐human Primate Research, Key Laboratory of CNS Regeneration (Ministry of Education), Guangdong‐Hongkong‐Macau Institute of CNS Regeneration Jinan University Guangzhou China; ^2^ Department of Neurology, Affiliated Hospital of North Sichuan Medical College Institute of Neurological Diseases, North Sichuan Medical College Nanchong China; ^3^ Department of Medical Imaging, First Affiliated Hospital Jinan University Guangzhou China; ^4^ Department of Medical Imaging, State Key Laboratory of Oncology in South China, Guangdong Provincial Clinical Research Center for Cancer Sun Yat‐sen University Cancer Center Guangzhou China

**Keywords:** myelination, MyRF, non‐human primate, oligodendrocytes, TDP‐43

## Abstract

Growing evidence indicates that non‐neuronal oligodendrocyte plays an important role in Amyotrophic lateral sclerosis (ALS) and other neurodegenerative diseases. In patient's brain, the impaired myelin structure is a pathological feature with the observation of TDP‐43 in cytoplasm of oligodendrocyte. However, the mechanism underlying the gain of function by TDP‐43 in oligodendrocytes, which are vital for the axonal integrity, remains unclear. Recently, we found that the primate‐specific cleavage of truncated TDP‐43 fragments occurred in cytoplasm of monkey neural cells. This finding opened up the avenue to investigate the myelin integrity affected by pathogenic TDP‐43 in oligodendrocytes. In current study, we demonstrated that the truncated TDP‐35 in oligodendrocytes specifically, could lead to the dysfunctional demyelination in corpus callosum of monkey. As a consequence of the interaction of myelin regulatory factor with the accumulated TDP‐35 in cytoplasm, the downstream myelin‐associated genes expression was downregulated at the transcriptional level. Our study aims to investigate the potential effect on myelin structure injury, affected by the truncated TDP‐43 in oligodendrocyte, which provided the additional clues on the gain of function during the progressive pathogenesis and symptoms in TDP‐43 related diseases.

## Graphical abstract

A proposed model of thedemyelination by pathological TDP‐43 in oligodendrocyte. The accumulation of truncated TDP‐43 inoligodendrocyte, could interact with MyRF in the cytoplasm, preventing itsnuclear localization and affecting the expression of myelin‐associated genes, therefore led to the dysfunctional demyelination in corpuscallosum.

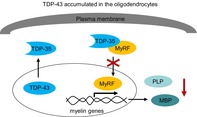

## 1 INTRODUCTION

TAR DNA/RNA binding protein 43 (TDP‐43) was the first identified protein in the postmortem spinal cord and cortex samples from amyotrophic lateral sclerosis (ALS) and frontotemporal lobar degeneration (FTLD) patients [1], then later found in the brains of patients with other neurodegenerative diseases, including Alzheimer's disease (AD), lewy body diseases, and Huntington's disease (HD) [2, 3, 4]. More than 50 mutations of TARDBP associated with ALS/FTLD have been identified, further supporting a causal role of TDP‐43 pathology [5, 6]. Two conserved RNA recognition motifs RRM1 and RRM2 in TDP‐43 render their nucleic acid‐binding activity [7, 8] to regulate different functions of gene transcription or RNA processing [7, 9, 10, 11]. TDP‐43 also contains a C‐terminal glycine‐rich domain to associate with a large number of proteins [9, 10, 12]. Typical TDP‐43 pathology is characterized by the abnormal formation of TDP‐43 cytosolic aggregations concurrent with loss of nuclear TDP‐43, which has led to hypotheses that both gain‐ and loss‐of‐function of TDP‐43 mutations can lead to the pathogenic outcomes [13, 14, 15, 16]. While current studies mainly focus on the suppressing normal TDP‐43 or artificial overexpressing TDP‐43 in nucleus to change genes' processing, but limited investigations on the potential influences of the TDP‐43 aggregations in cytoplasm. Recently, we reported that, in the neural cells of non‐human primate brains, the truncated TDP‐43 fragments were emerged and localized in cytoplasm to form aggregates, consisting of highly ubiquitinated, phosphorylated, and cleaved TDP‐43 forms [17, 18], which are similar to those seen in patient brains [1, 10, 19, 20, 21, 22]. This finding opened up a new avenue to investigate the mechanism on myelin integrity mediated by TDP‐43 in oligodendrocytes.

Growing evidence indicates that non‐neuronal oligodendrocyte plays an important role in ALS/FTLD and other neurodegenerative diseases. In terms of TDP‐43 associated diseases, the oligodendrocyte morphological defects can be seen and accompanied by abnormal myelin sheath structure and large number of inclusions in patients [23, 24, 25, 26, 27], and even the damaged myelin was seen in the differentiated NG2+ precursor cells in vitro [23]. Through the pathological detection of intracellular aggregates, TDP‐43 is positively stained in oligodendrocytes and also exhibits characteristics of the cytoplasmic accumulation similar to the neuronal cells [28, 29, 30, 31, 32, 33, 34]. Such as in the anterior horn region of the spinal cord in sporadic ALS cases [35], the ALS‐related FOSMN syndrome patients brain [36], the deep cortical white matter region of FTLD‐TDP brain [25], and even the oligodendrocytes of AD and Lewy body dementia [37], the cytoplasmic TDP‐43 accumulation could be detected. However, whether and how the accumulation of TDP‐43 in oligodendrocytes affects the myelin structures and neuronal cytotoxicity is rarely elucidated. To better explore thoroughly, we demonstrated that the presence of truncated TDP‐43 fragments in cytoplasm of oligodendrocytes specifically, could impair the axonal integrity through demyelination in the monkey corpus callosum. Furthermore, we found that the truncated C‐terminal TDP‐43 fragments bound the myelin regulatory factor (MyRF) in the cytoplasm, preventing its nuclear localization and affecting the expression myelin‐associated genes. This study aims to investigate the effect and mechanism of the pathological TDP‐43 in oligodendrocyte, which underlies additional clues for explaining the progressive gain of abnormal function of TDP‐43 on non‐neuronal toxicity.

## 2 MATERIALS AND METHODS

### 2.1 Plasmids, viruses, and antibodies

The cDNAs encoding full‐length human mutant TDP‐43 (M337V) and truncated C‐terminal TDP‐(77–414) (M337V) (TDP‐35(M337V)) were subcloned into the AAV9‐MBP vector to generate the AAV‐MBP‐TDP‐43 and ‐TDP‐35 vectors. The control AAV‐GFP vector consists of the same vector and promoter. The titers of the virus of AAV‐MBP‐TDP‐43, ‐TDP‐35, and ‐GFP, are 1 × 10^12^ vg/mL (Vigene Biosciences, Inc, China). The primary antibodies were used as following: anti‐FLAG (Proteintech, 10782‐2‐AP), anti‐NeuN (Abcam, ab177487), anti‐GFAP (Abcam, ab7260), anti‐IBA1 (Abcam, ab178847), anti‐Oligo2 (Sigma, AB9610), anti‐TDP‐43(C‐terminal to the fusion protein Ag4003) (Proteintech, 12892‐1‐AP), anti‐TDP‐43(C‐terminal corresponding to the residues surrounding Gly400 of human TDP‐43) (Cell Signaling, 3448S), anti‐TDP‐43(N‐terminal to the residues 1–89 amino acids of human TDP‐43) (Santa Cruz, sc‐376311), anti‐TDP‐43 (N‐terminal corresponding to residues surrounding Val47 of human TDP‐43) (Cell Signaling, E2G6G), anti‐MBP (Abcam, ab21801), anti‐PLP1 (Bio‐Rad, 159401), anti‐MyRF(N‐terminal corresponding to the cytoplasmic N‐terminal chain of human MyRF) (Sigma, ABN45), anti‐MyRF(toward the N‐terminal region of human C11orf9) (Thermo Fisher, PA5‐68638), anti‐GST (Invitrogen, MA4‐004), anti‐Vinculin (Sigma, MAB3574), anti‐GAPDH (Proteintech, 60004‐1‐Ig) and anti‐Tubulin (Proteintech, 66240‐1‐Ig). All secondary antibodies were purchased from Boster Bio and Thermo.

### 2.2 Animals

The wild‐type C57BL/6 mice at 6–10 months of age (*n* = 8 each group, male mice per group) and the WT cynomolgus monkeys aged 8–12 years (*n* = 4 each group, male monkeys per group) were used in the study. Cynomolgus monkeys were raised at Guangdong Landau Biotechnology Co. Ltd., which is an Association for Assessment and Accreditation of Laboratory Animal Care‐accredited facility. Water, temperature, and humidity in the home cage in which monkeys were kept were closely monitored. Monkey's health was daily examined by veterinarians. Mice were kept at the animal facility at Jinan University. Approval from the local ethical committee of Jinan University for animal experiments was obtained. All methods were carried out in accordance with relevant guidelines and regulations. This study was also carried out in compliance with the ARRIVE guidelines. For the randomization, on arrival from the animals' facilities, each animal was assigned a temporary random number within the weight range. Then the animals were randomly divided into two experimental groups for the injection: the control group and the target group. For each group, a cage was selected randomly from the pool of all cages, in which the animals were given their permanent numerical designation.

### 2.3 Ethics approval

All animal‐related experiments followed the NIH guide for the care and use of laboratory animals. The protocols were approved in advance by the Animal Care and Use Committee of Guangdong Landau Biotechnology Co. Ltd and Jinan University. This study occurred in strict compliance with the “Guide for the Care and Use of Laboratory Animals of the Institute of Laboratory Animal Science (est. 2006)” and “The use of non‐human primates in research of the Institute of Laboratory Animal Science (est. 2006)” to ensure the safety of personnel and animal welfare.

### 2.4 Cell culture and transfection

The human embryonic kidney (HEK) 293 cell line was purchased from ATCC and cultured in DMEM/F12 medium, containing 10% FBS, 100 U/mL penicillin, 100 μg/mL streptomycin (Thermo Fisher Scientific) and 0.25 μg/mL amphotericin B (Thermo Fisher Scientific). The cells were maintained at 37°C in 5% CO_2_ incubators. The growth medium was refreshed every 2 days. For transient transfection, cells were plated at 70%–80% confluence and transfected with plasmid DNA by using Lipofectamine 3000 (Invitrogen) for 24–72 h.

### 2.5 Stereotaxic injection

For mouse brain injection, the WT C57BL/6 mice at 6–10 months of age (*n* = 8 and male mice per group) were injected with AAV virus and sacrificed after 1 month surgery. In detail, the mice were anesthetized with an i.p. injection of 2.5% Avertin, and their heads were placed in a head stereotaxic frame (RWD Life Science) equipped with a digital manipulator, a UMP3‐1 Ultra pump, and a 10 μL Hamilton microsyringe. The 33G needle was inserted through a 1 mm burr hole in the scalp. Injections occurred at the following stereotaxic coordinates: 0.6 mm prior to bregma and 2.5 mm ventral to the dura, with bregma set at zero, abiding by the midline, while 1.5 mm lateral to the midline for the lateral corpus callosum. The microinjections were carried out at the rate of 0.2 μL/min. A total of 0.5 μL of the target virus was injected into different locations of the right corpus callosum of mouse. The microsyringe was left in place for an additional 10 min before and after each injection. For monkey brain injection, the WT cynomolgus monkeys aged 8–12 years (*n* = 4 and male monkeys per group) were injected of AAV virus and sacrificed after 2 months surgery. As the previous report [18], each monkey was anesthetized by intraperitoneal injection of 0.3–0.5 mg atropine, followed by 10–12 mg ketamine and 15–20 mg pentobarbital columbarium per kg body weight. Monkeys were held steady on a stereotaxic apparatus (RWD Life Science). The exact location of the corpus callosum to receive the stereotaxic injection was determined by MRI before injection. A total of 10 μL of the target virus was injected into different locations of the right corpus callosum of monkey, while the AAV‐GFP control was injected into the opposite side of the same monkey. The needle insertion depth was calculated based on the pre‐surgery MRI. After 8 weeks of injection, their brain tissues were isolated for immunohistochemical and Western blotting analysis. As the stereotaxic injection expressed the virus specifically in a limited brain region, the tissue harvest was according to the injection sites and needle pathway. The same animal tissue (monkey and mouse) was used for both Western blotting and PCR experiments to keep the consistency of the samples.

### 2.6 MRI and image analysis

MRI image acquisition monkeys were anesthetized by ketamine (10 mg/kg, i.m.) and placed into the scanner in the prone position for Ax CUBE T2 three‐dimensional MR scan. The scans were collected on a 3.0 T MR machine (GE 750) using a custom‐designed 8‐channel radio‐frequency surface head coil at Jinan University. We performed T2 sequence, with the following parameters: TR = 9.5 ms, TE = 4.0 ms, slice thickness = 0.5 mm, slice per slab = 196, matrix size = 256 × 256 mm, FOV = 150 × 150 mm, and voxel size = 0.5 × 0.5 × 0.5 mm. The analysis of each ROI (Region of Interest) was performed using the voxel‐based raw data, and the cumulative frequency distributions of 10th, 90th, median, mean, skewness (skew), and kurtosis were calculated. ROI‐based enhancement was also analyzed.

### 2.7 RNA‐Seq analysis

We previously used stereotaxic injection of AAV9 expressing transgenic human TDP‐43(M337V) under the UBC promoter into the brains of male monkeys at the age of 8–12 years [18, 38]. Two months after injection, the injected brain tissues were isolated and preserved in liquid nitrogen for transcript sequencing. The RNA library was extracted from monkey's white matter using TRIzol Reagent according to the manufacturer's protocol. Construction of the cDNA library and sequencing were performed by NOVOGENE Co., Ltd. (Beijing, China). Clean reads were obtained after discarding reads containing adapter, poly‐N, or low‐quality raw data. High‐quality reads were aligned to the existing reference genome. Next, the HTseq was used to convert aligned short reads into read counts. The differentially expressed genes (DEGs) were chosen according to the criteria of a fold change of greater than 1.3 and an adjusted *p*‐value of less than 0.05.

### 2.8 Immunofluorescence and immunohistochemistry

Mice and monkeys were anesthetized with 5% chloral hydrate and perfused with 0.9% NaCl, followed by 4% paraformaldehyde (PFA). The brains were removed and post‐fixed in 4% PFA overnight at 4°C. The brains were transferred to 30% sucrose for 48 h and then cut into 15‐ or 30‐μm sections with the cryostat (Leica CM1850) at −20°C. Sections were blocked in 4% donkey serum with 0.2% Triton X‐100 and 3% BSA in PBS for 1 h. For immunofluorescent staining, 15‐μm sections were incubated with primary antibodies in the same buffer at 4°C overnight. The concentration of the antibody for immunohistochemistry was as following: anti‐FLAG (1:3000), anti‐NeuN (1:1000), anti‐GFAP (1:5000), anti‐IBA1 (1:500), anti‐Oligo2 (1:1000), anti‐TDP‐43 (C‐terminal, Proteintech, 12,892‐1‐AP) (1:2000), anti‐MyRF (N‐terminal, Sigma, ABN45) (1:100). After washing with PBS, the sections were incubated in fluorescent secondary antibodies. Fluorescent images were acquired with a Zeiss microscope (Carl Zeiss, Axiovert 200 MOT) and either a 40× or 63× lens (LD‐Achroplan 40×/0.6 or 63×/0.75) with a digital camera (Hamamatsu, Orca‐100) and Openlab software (Improvision). For immunohistochemistry staining, after blocking, 30‐μm sections were incubated with antibodies for at least 48 h at 4°C. A biotin/avidin immune assay (Vector Laboratories) and DAB kit (Invitrogen, 00‐2020) were used. Images were acquired with a Zeiss microscope (Carl Zeiss, Imager A.2) and either a 40× or 63× lens (Plan‐Apochromat 40×/0.95 or 63×/1.4) with a digital camera (Carl Zeiss, AxioCam HRc) and AxioVision software.

### 2.9 Electron microscopy

For the electron microscopic study, the freshly isolated monkey or mice brain tissue blocks were fixed with 3% glutaraldehyde and 1% osmium tetroxide for 24–48 h, then dehydrated step by step with 30%, 50%, 70%, 80%, 90%, 95%, and 100% acetone. The acetone was gradually replaced with an embedding agent and the samples were embedded in Eponate 12 (Ted Pella, Redding, CA). Ultrathin sections (60 nm) were cut using a Leica Ultracut S ultramicrotome. The sections placed on the copper mesh were stained with uranium acetate at room temperature for 10–15 min and then stained with lead citrate for 1–2 min. A Hitachi (Tokyo, Japan) H‐7500 electron microscope was used for the ultrathin section examination. The axon and myelin fiber diameters were measured using ImageJ (NIH). In general, the G‐ratios, which were calculated and plotted against axon diameter with linear regression, were shown after treatment, when compared with vehicle group. More than 100 axons were examined and calculated in each group.

### 2.10 Western blotting, Q‐PCR and immunoprecipitation

To assess protein and gene expression levels, the brain tissues were crushed using a soluble fraction buffer that contained 10 mM Tris (pH 7.4), 100 mM NaCl, 1 mM EDTA, 1 mM EGTA, 0.1% SDS, and 1% Triton X‐100, supplemented with protease inhibitors (Sigma, P8340). The lysates were then mixed with 1× SDS sample buffer and sonicated for 15 s after being incubated at 100°C for 10 min. The Western blotting approach utilized Tris‐glycine gels to load the total lysates (10–20 μg), which were then transferred onto nitrocellulose membranes. The ECL Prime Chemiluminescence kit (GE Healthcare Amersham) was used for the development. The same animal was used for both western blotting and PCR experiments to keep the consistency of the monkey and mouse samples. The Q‐PCR analysis required 1.5 μg of RNA taken from the Superscript III First‐Strand Synthesis System (Invitrogen), in order to facilitate the reverse transcription reactions. In a 20 μL reaction, 1 μL of cDNA was mixed with each primer and 10 μL SYBR Select Master Mix (Applied Biosystems). The reaction was undertaken on a real‐time thermal cycler (Eppendorf, Realplex Mastercycler). The primer for the conserved sequences on monkey and mouse‐associated genes are as follows:

MBP‐forward 5′‐AGTACCTGGCCACAGCAAGT‐3′.

MBP‐reverse 5′‐AGGATGCCCGTGTCTCTGT‐3′.

MOG‐forward 5′‐TGCCCTGCTGGAAGATAAC‐3′.

MOG‐reverse 5′‐AGCCAGTTGTAGCAGATGAT‐3′.

PLP1‐forward 5′‐CTGCCTCTTTCTTCTTCC‐3′.

PLP1‐reverse 5′‐GATGGTGGTCTTGTAGTCG‐3′.

GAPDH‐forward 5′‐TGTCAAGCTCATTTCCTGGT‐3′.

GAPDH‐reverse 5′‐TCTTACTCCTTGGAGGCCATG‐3′.

For immunoprecipitation, a total of 500 μg brain tissues in cold 1% NP‐40 buffer (50 mM Tris, pH 7.4, 50 mM NaCl, 0.1% Triton X‐100, and 1% NP‐40 with 1× protease inhibitors and 100 μM PMSF) were precleared with protein A agarose beads (Sigma, P1406), then the samples were immunoprecipitated by anti‐FLAG at 4°C overnight. Protein A agarose beads were added to capture the target proteins for 2 h at 4°C. Ice‐cold lysis buffer was used to wash beads three times. Proteins from the compound and inputs were subjected to Western blotting and Coomassie brilliant blue R‐250 staining.

### 2.11 In vitro GST‐pull‐down assay

The purified GST‐TDP‐35 linked on sepharose beads were diluted in ice‐cold assay buffer (25 mM Tris‐HCl, 10 mM MgCl_2_, 100 lg/mL purified rabbit creatine kinase, 50 mM phosphocreatine, 1 mM ATP, pH 7.6). Mouse or monkey tissues from the corpus callosum were homogenized at 1 g/mL in ice‐cold assay buffer using 20 strokes of a glass Dounce homogenizer, and the homogenates were centrifuged at 500× *g* for 5 min at 4°C to pellet unbroken tissues and membranes. The supernatant was collected and stored on ice while protein concentrations were determined using a BCA Protein Assay Kit (Thermo Scientific/Pierce). The lysates (200 μL) at 0.5 mg protein/mL were incubated with GST‐TDP‐35 beads (200 μL) at 37°C for 1 h with shaking at 300 rpm. The beads were centrifuged and combined with the protein loading dye (0.2% SDS) for SDS‐PAGE and Western blot analysis using the anti‐MyRF or anti‐GST antibody.

### 2.12 Dual‐luciferase reporter assay

The mouse MBP promoter was subcloned into the pGL3‐Basic vector for luciferase experiments. The human HEK293 cell line was co‐transfected of pGL3‐MBP‐luci with PRK‐MyRF, ‐TDP‐35, and ‐TDP‐△NLS (NLS deleted on full‐length TDP‐43, as the positive control of its cytoplasmic localization) respectively. After 48 h, the cells were harvested and lysed with lysis buffer. Each sample was divided into duplicates, one half was lysed in 1% NP‐40 buffer, and the protein levels were determined via Western blotting. The remaining half was used for the reporter assay using Luciferase Assay System (Promega, E1910). The firefly luciferase activity of the MBP promoter construct was normalized against the Renilla luciferase output of the co‐transfected pGL3‐T5 vector.

### 2.13 Nuclear and cytoplasmic protein extraction

The brain tissues of WT and HD KI mice were harvested and separated nuclear and cytoplasmic proteins. Nuclear and cytoplasmic protein extraction was conducted using the Nuclear and Cytoplasmic protein extraction Kit (P0027, Beyotime), according to the manufacturer's instructions, and then analyzed using western blotting.

### 2.14 Statistical analysis

Results are expressed as mean ± SEM. Prism9 (GraphPad Software) was used for statistical analysis, based on the Shapiro–Wilk test to conduct a goodness‐of‐fit analysis of the normal distribution. When the experimental groups (*n* = 3 or 4 samples per group) were compared, Tukey's *t*‐test was used to calculate statistical significance. For all the experiments, statistical significance was calculated using one‐way ANOVA, followed by Tukey's multiple comparisons tests. A *p*‐value <0.05 was considered as significant. The statistical detail including the “*p*” values, degrees of freedom (df), and “*t*” values was included in the related figure legends.

## 3 RESULTS

### 3.1 The cleaved TDP‐43 fragment leads to demyelination in oligodendrocyte of monkey specifically

We previously reported that the ubiquitous expression of transgenic human TDP‐43 (M337V) under the UBC promoter in the monkeys motor cortex could lead to the neuronal toxicity with pathological accumulation of TDP‐43 in the cytoplasm [18, 38]. The emerging issue is whether the mis‐localized TDP‐43 elicits the other neuropathology such as myelin integrity and axon degeneration. We isolated white matter, in which glial cells are enriched, from the injected monkey brains to investigate the high‐throughput transcriptome profiling mediated by TDP‐43 (M337V) in glial cells. Volcanic map analysis showed the DEGs by the general expression of TDP‐43 (M337V) and GFP in the white matter of monkey under the UBC promoter (Figure S1A). The cortical TDP‐43 (M337V) expression had 1559 up‐regulated, 1523 down‐regulated, and 22,327 unchanged genes respectively, when compared with GFP controls in monkey (Figure S1A). The enriched DEGs with “Axon guidance” by KEGG pathway were clustered obviously after TDP‐43 (M337V) expressing in the white matter (Figure S1B). According to the visual heatmap of the embraced DEGs, some relative “axon guidance”‐associated genes were dysregulated in the TDP‐43 (M337V) expressing tissues of monkey (Figure S1C).

**FIGURE 1:**
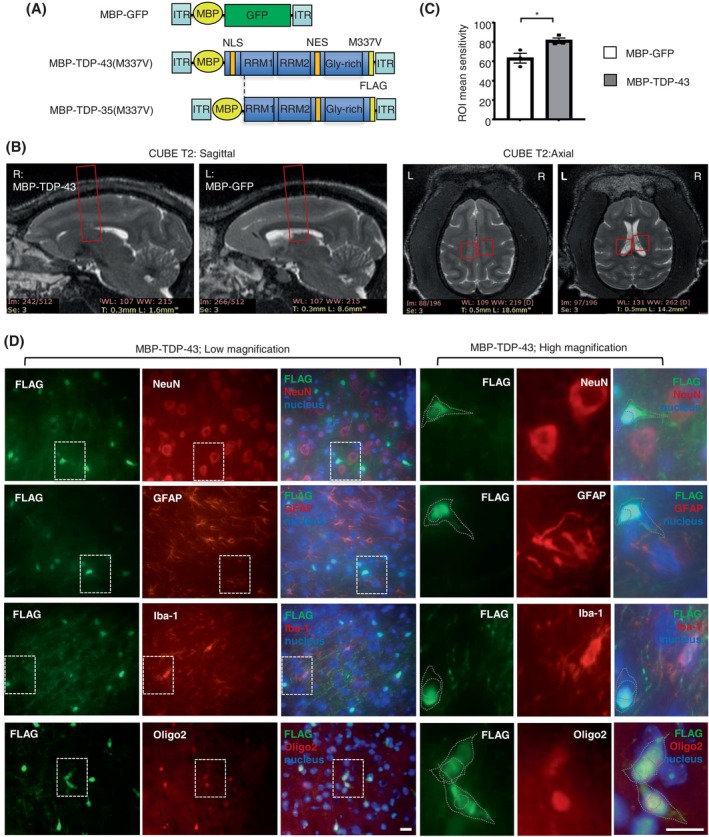
The specific expression of mutant TDP‐43 in oligodendrocytes of the monkey brain. (A) Schematic diagram for the generation of adeno‐associated virus, under the MBP promoter, which expresses the mutant TDP‐43, truncated TDP‐35 lacking the NLS, or GFP control vectors. The mutant TDP‐43 was injected into the right corpus callosum of the male wild‐type monkey aged 8–12 years. The opposite side of the same monkey was injected with GFP control. MBP, myelin basic protein; NES, nuclear export signal; NLS, nuclear import signal; RRM: RNA recognition motifs. (B) Magnetic resonance imaging (MRI) analysis revealed the monkeys' sagittal and axial images. The boxed areas indicate the injected regions in vivo. The injected right side of the corpus callosum by AAV‐MBP‐TDP‐43 showed the impairment of the reduced density in the boxed areas images, as compared with the opposite left side injected with AAV‐MBP‐GFP control. (C) The analysis of each ROI (region of interest) was performed using the voxel‐based raw data, and the cumulative frequency ROI‐based mean enhancement was analyzed, based on the minimum, maximum, median, 10th percentile, and 90th percentile values. (*n* = 3, male Cynomolgus macaques of 8–12 years old for 2 months injection on corpus callosum). One‐way ANOVA followed with Tukey's test. *p = 0.0338 (*t* = 3.172, df = 4). Data are mean ± SEM. (D) The left panels indicated the double immunofluorescent staining of the monkey corpus callosum injected with AAV‐MBP‐TDP‐43, using the antibodies to FLAG (green) with NeuN, GFAP, Iba‐1 or Oligo2 (red), respectively. The nuclei were stained with DAPI (blue). Representative images were obtained from three male 8–12‐years‐old monkeys. The different cellular distributions of FLAG without the typical markers of NeuN, GFAP, and Iba‐1, indicated that the expressed exogenous TDP‐43 did not into the neuronal cell, astrocyte, and microglia respectively. The significant co‐localization of FLAG with the oligodendrocyte marker (Oligo2) showed the successfully and specifically expression of TDP‐43(M337V) in the same oligodendrocyte of monkey brain. The right panels showed the high magnification of the immunofluorescent staining in the white box of the left panel (the low magnification), in which, the cytoplasmic distribution of the exogenous TDP‐43 with Oligo2 was observed in monkey corpus callosum. Scale bar: 10 μm.

**FIGURE 2:**
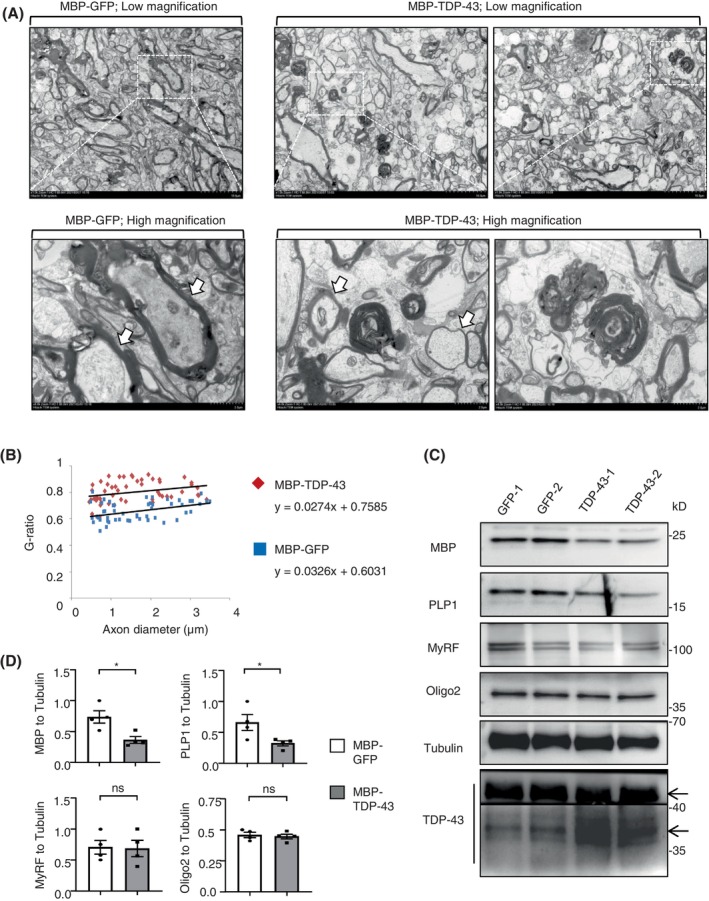
The cleaved TDP‐43 in oligodendrocyte leads to demyelination in monkey brain. (A) Electron microscopy images showed that the injection of AAV‐MBP‐TDP‐43 had obvious demyelinated or degenerated axons, as compared with AAV‐MBP‐GFP control (male Cynomolgus macaques of 8–12 years old for 2 months). Scale bar: 0.5 μm (low magnification) and 0.1 μm (high magnification). (B) G‐ratios were calculated and plotted against the axon diameter with linear regression. The *g*‐ratio was significantly deteriorated by the expression of TDP‐43 (M337V) in monkey brain (*g* = 0.0274 ± 0.7585) compared to the GFP control (*g* = 0.0326 ± 0.6031). At least 100 axons per genotype were examined (*n* = 3, 8–12‐year‐old male cynomolgus macaques 2 months after injection in the corpus callosum). (C) Western blotting of the anti‐TDP‐43 shows that the expressing TDP‐43(M337V) promoted the cleavage of TDP‐35 fragments, and led to the significant reduction of the myelin‐associated proteins of MBP and PLP1, but no alter the expression of the total MyRF and Oligo2. Tubulin served as a loading control (male cynomolgus macaques of 8–12 years old for 2 months). (D) Quantitative analysis of the band intensity ratios of MBP, PLP1, MyRF, and Oligo2 to Tubulin in panel (C). The data are presented and obtained from 4 independent experiments. (*n* = 4, male cynomolgus macaques of 8–12 years old that had been injected in the corpus callosum for 2 months). One‐way ANOVA followed with Tukey's test. MBP *p = 0.0169 (*t* = 3.277, df = 6). PLP1 *p = 0.0476 (*t* = 2.484, df = 6). ns: not significant. Data are mean ± SEM.

However, the alternation of these DEGs in “axon guidance” could directly or indirectly impair the axon guidance, synaptogenesis, progenitor dynamics, and cell migration through various mechanisms. Therefore, it is crucial to investigate the specific effect of the pathological TDP‐43 in oligodendrocytes. To address this, we generated the MBP (myelin basic protein) promoter‐based AAV vectors capable of selectively driving gene expression in oligodendrocytes. Subsequently, we injected AAV‐MBP‐TDP‐43 into the right corpus callosum of wild‐type monkeys aged 8–12 years. The opposite side of the same monkey was injected with AAV‐MBP‐GFP control (Figure 1A). After 2 months, the magnetic resonance imaging (MRI) analysis revealed the impairment of the reduced density in the injected areas by AAV‐MBP‐TDP‐43, as compared with the opposite side of AAV‐MBP‐GFP control (Figure 1B), in which of the ROI (Region of Interest) the mean distribution was quantified based on the minimum, maximum, median, 10th percentile, 90th percentile, median, skewness (skew) and kurtosis values of the monkey's brain (Figures 1C and S2). The double immunofluorescent staining indicated the cellular distributions of the exogenous TDP‐43 (M337V) without any typical markers of the neuronal cell (NeuN), astrocyte (GFAP), and microglia (Iba‐1), but with the significant oligodendrocyte marker (Oligo2), which showed the successfully and specifically expression of TDP‐43 (M337V) in the oligodendrocytes of monkey brain (Figure 1D, left panel). Furthermore, in the same oligodendrocyte, the expressed exogenous TDP‐43 (M337V), labeled as FLAG, was mis‐localized in the cytoplasm of monkey brain in vivo (Figure 1D, right panel and Figure S3A). Then the electron microscopy was performed to show the obvious demyelinated or degenerated axons by the TDP‐43 (M337V) accumulation (Figure 2A, B). Western blotting (Figures 2C, D and S3B) showed that the expressing TDP‐43 (M337V) occurred the cleavage of TDP‐35 fragments, and led to the significant reduction of the myelin‐associated proteins of MBP and PLP1, at the transcriptional level, but not alter the expression of MyRF.

### 3.2 Truncated TDP‐35 in oligodendrocyte also leads to the demyelination of the mouse brain

**FIGURE 3:**
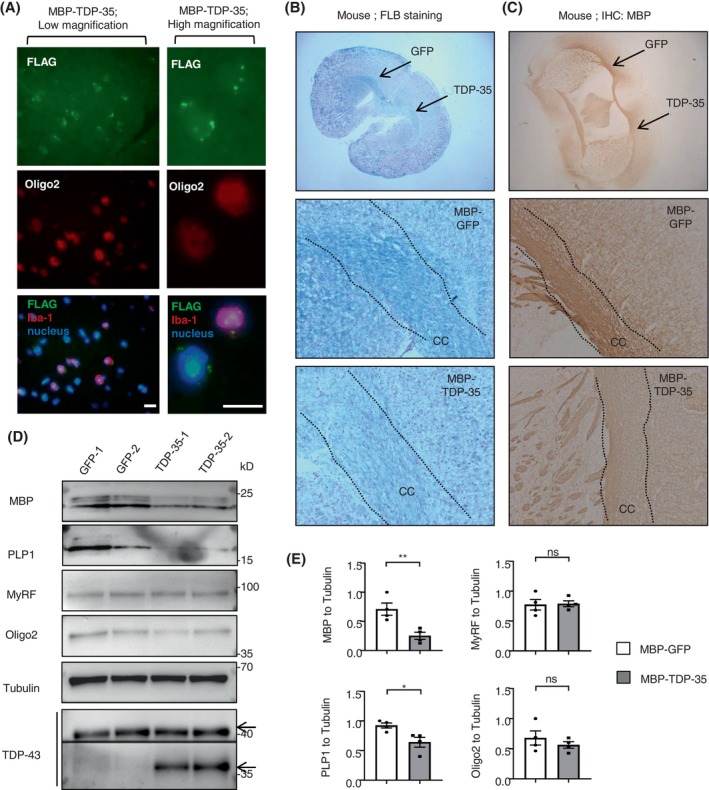
Truncated TDP‐35 expressed in oligodendrocytes of mice leads to the demyelination. (A) Immunofluorescent staining of the AAV‐MBP‐TDP‐35 injected mouse brain, using antibodies to Oligo2 (red) and FLAG (green). The nuclei were stained with DAPI (blue). Representative images were obtained from three 6‐10‐month‐old mice. Immunofluorescent staining of mouse brains showed the clear co‐localization of TDP‐35 with the oligodendrocyte marker (Oligo2) in the left panel (low magnification). Magnification of immunofluorescent staining in the white box showed the co‐localization of Oligo2 with the truncated TDP‐35 indicated by FLAG in the cytoplasm in the right panel (high magnification) (6–10 months old male mice 1 month after injection in the corpus callosum). Scale bar: 10 μm. (B) The luxol fast blue (LFB) myelin staining, showed that the injection of AAV‐TDP‐35 causes the loose of myelin density in mouse corpus callosum (CC), as compared with the opposite side expression of GFP control virus. Scale bar: 200 μm (low magnification) and 40 μm (high magnification) (male mice of 6–10 months old for 1 month injection in the corpus callosum). (C) Immunohistochemical staining with MBP antibody, indicated that the injection of AAV‐TDP‐35 causes demyelination in mouse corpus callosum (CC), as compared with the opposite side expression of GFP control virus. Scale bar: 200 μm (low magnification) and 40 μm (high magnification). (D) Western blotting with the anti‐C‐terminal TDP‐43 showed that the accumulation of the truncated TDP‐35 fragment led to the reduction of myelin‐associated protein levels of MBP and PLP1, from the 6–10‐month‐old mice after AAV injection for 1 month, without any change on the MyRF and Oligo2 level, was observed in the truncated TDP‐35 expressed corpus callosum in mouse brain (male mice of 6–10 months old for 1 month injection in the corpus callosum). (E) Quantitative analysis of the band intensity ratios of MBP, PLP1, MyRF, and Oligo2 to Tubulin. The data are obtained from 4 independent experiments (*n* = 4 male mice at 6–10 months of age 1 month after injection in the corpus callosum). One‐way ANOVA followed with Tukey's test. MBP **p = 0.0089 (*t* = 3.807, df = 6). PLP1 *p = 0.0230 (*t* = 3.032, df = 6). ns: not significant. Data are mean ± SEM.

To explore whether the mis‐localized TDP‐43 aggregates in cytoplasm of oligodendrocytes affected the myelin integrity, we also generated AAV9 vector expressing the truncated TDP‐35 under the MBP promoter. Since TDP‐35 lacks NLS, its cytoplasmic distribution can result in the cytoplasmic toxicity in oligodendrocytes. We then injected the AAV‐MBP‐TDP‐35 or ‐GFP control into the oligodendrocyte‐enriched corpus callosum of mouse brain at 6–10‐months of age (Figure 1A). First, four kinds of N‐terminal or C‐terminal TDP‐43 antibodies were used to confirm that the loss of NLS was only detected by the C‐terminal, but not N‐terminal antibody, in the transfected HEK293 (Figure S4A) or the injected mouse brain (Figure S4B). Similar to the monkey brain, the clear co‐localization of exogenous TDP‐35 with Oligo2, which indicated by FLAG or TDP‐43 antibodies in cytoplasm (Figures 3A and S5A). Electron microscopy also revealed the obvious demyelination or axonal degeneration in the mouse brain, affected by the MBP‐TDP‐35 expression in oligodendrocyte specifically (Figures S6A and S6B). Through the luxol fast blue myelin staining (Figure 3B), or immunohistochemical staining with MBP antibody (Figure 3C), the injection of AAV‐MBP‐TDP‐35 causes the significant reduction of myelin morphological structures, in the injected region of mouse corpus callosum, when compared with the opposite side of GFP control. Western blotting also showed that in mouse brain in vivo, the accumulation of truncated TDP‐35 led to the reduced myelin‐associated protein of MBP and PLP1, but without any change on total MyRF level (Figures 3D, E and S5B). Oligodendrocytes and myelin abnormalities can slow or stop fast axon transport, resulting in synaptic loss or axonal degeneration [39]. Therefore, the idea that the pathological TDP‐43 affects the expression of selective genes in mature oligodendrocytes is supported by the fact that the truncated TDP‐43 co‐localized with OLIG2 specifically, but did not affect its expression.

### 3.3 The interaction of the truncated TDP‐35 with MyRF affects the myelin genes dysregulation

**FIGURE 4:**
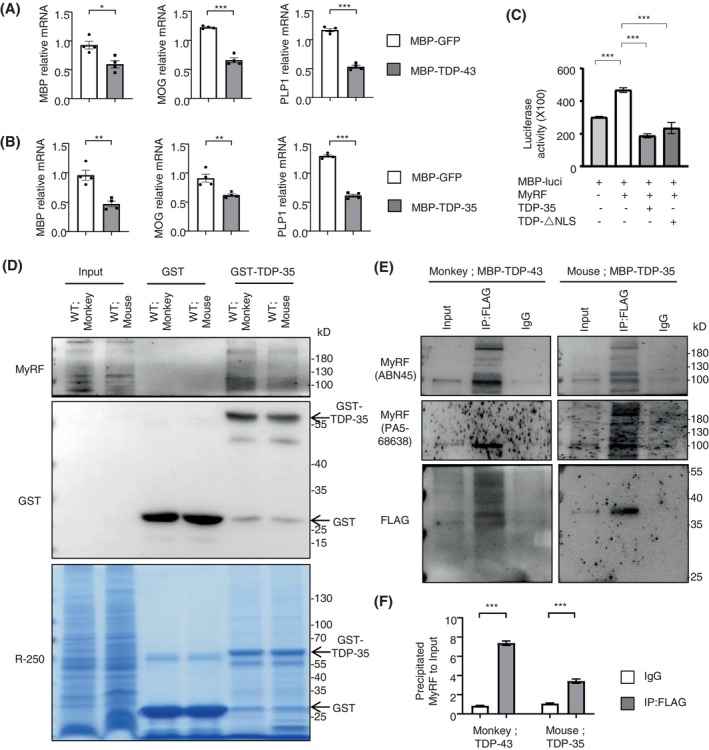
The interaction of truncated TDP‐43 with MyRF in cytoplasm of monkey and mouse brain. (A) Quantitative real‐time PCR analysis of the mRNA expression of *MBP*, *MOG*, and *PLP1*, in the AAV‐MBP‐TDP‐43 injected monkey brain, as compared with AAV‐MBP‐GFP control (*n* = 4, male cynomolgus macaques of 8–12 years old for 2 months injection in the corpus callosum). One‐way ANOVA followed with Tukey's test. MBP *p = 0.0112 (*t* = 3.615, df = 6). MOG ***p<0.001 (*t* = 12.86, df = 6). PLP1 ***p<0.001 (*t* = 16.70, df = 6). Data are mean ± SEM. (B) The mRNA expression of *MBP*, *MOG*, and *PLP1*, in the AAV‐MBP‐TDP‐35 injected mouse brain, as compared with AAV‐MBP‐GFP control, by the qPCR analysis (*n* = 4 per group, male C57BL/6 mice at 6–10 months of age for 1 month injection in the corpus callosum). One‐way ANOVA followed with Tukey's test. MBP **p = 0.0024 (*t* = 5.023, df = 6). MOG **p = 0.0074 (*t* = 3.965, df = 6). PLP1 ***p < 0.001 (*t* = 20.92, df = 6). Data are mean ± SEM. (C) The luciferase reporter vector of human MBP promoter was co‐transfected with MyRF, TDP‐35, and TDP‐△NLS to assess its transcription activity in HEK293 cell line in vitro. Performing the luciferase assay, the expression of MyRF markedly promotes the MBP promoter activity, while the co‐expression of TDP‐35 significantly reduces this luciferase reporter activity. The co‐transfection of TDP‐△NLS (deletion of the NLS on full‐length TDP‐43 sequence) was used as the positive control, as the consequence of the cytoplasmic distribution of TDP‐43. The data are obtained from four independent experiments. One‐way ANOVA followed with Tukey's test. MyRF with or not ***p = 0.0008 (*t* = 11.53, df = 6). TDP‐35 with or not ***p < 0.001 (*t* = 15.37, df = 6). TDP‐△NLS with or not ***p<0.001 (*t* = 6.22, df = 6). The data are presented as mean ± SEM. (D) The GST‐TDP‐35 and GST fusion proteins were purified and incubated with white matter tissues from monkeys and mice brains, the in vitro pulldown assay was detected using the antibodies against MyRF and GST, which showed that the TDP‐35 (fused with GST protein) could bind with MyRF directly when compared with the GST control. The Coomassie brilliant blue R‐250 staining displayed the equivalently purified proteins of GST and GST‐TDP‐35 basically. (E) Western blotting analysis of the immunoprecipitated FLAG labeled TDP‐43 or TDP‐35 in the injected monkey or mouse brain. Note that the expressed truncated TDP‐43 or TDP‐35 could bind the endogenous N‐terminal MyRF significantly in both tissues, using two kinds of N‐terminal MyRF antibodies. The input or IgG groups were served as controls (male mice of 6–10 months old or male monkeys of 8–12 years old for the injection in the corpus callosum). (F) Quantitative analysis of the band intensity ratios of the precipitated N‐terminal MyRF to input in monkey and mouse white matter tissue from four independent experiments. One‐way ANOVA followed with Tukey's test. TDP‐43 ***p<0.001 (*t* = 26.33, df = 6). TDP‐35 ***p<0.001 (*t* = 10.18, df = 6). The data are presented as mean ± SEM.

On the basis of aforementioned findings, we went on to test how the reduction of the myelin proteins by pathological TDP‐43. First, we performed the Q‐PCR to confirm that, the expressing TDP‐43 or TDP‐35 led to the significant reduction of the myelin‐associated proteins of MBP, MOG, and PLP1 at the transcriptional level, in the injected monkey (Figure 4A) and mouse (Figure 4B) brain respectively. To address the issue of myelin genes dysregulation by the truncated TDP‐43, we isolated the promoter region of the mouse MBP gene [40] and linked this promoter with the luciferase reporter. Through the luciferase assay, MyRF significantly increased the MBP promoter activity, which was inhibited by truncated TDP‐35 or TDP‐△NLS, in which the deletion of the NLS on full‐length TDP‐43 sequence would lead to the cytoplasmic distribution of TDP‐43 (Figure 4C). In previous report, another neurodegenerative aggregates protein of mutant Huntingtin, could abnormally binds MyRF, to affect its critical function for the regulation of transcription on myelin‐associated genes [41]. For this, we performed in vitro GST pulldown assay (Figure 4D) and in vivo immunoprecipitation (Figure 4E) of the TDP‐35 fragments from the white matter tissues. The results showed that the mutant TDP‐43 in monkey or the truncated TDP‐35 in mouse could interact with the N‐terminal MyRF directly, which was confirmed by two kinds of MyRF antibodies (Figure 4E, F).

**FIGURE 5:**
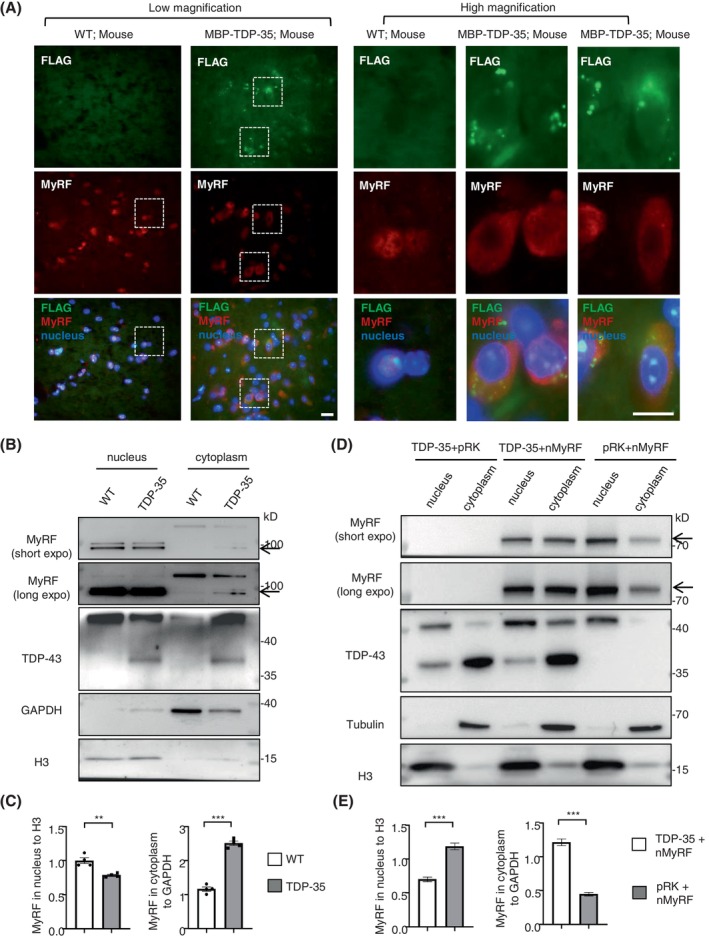
The increased cytoplasmic MyRF by truncated TDP‐43 in the mouse brain. (A) Immunofluorescent staining of the expressed truncated TDP‐35 in the oligodendrocyte of mouse brain, using the antibodies to FLAG (green) and MyRF (red), as compared with the wild‐type (WT) mouse. The nuclei were stained with DAPI (blue). Representative images were obtained from the aforementioned male 6–10‐month‐old mice. The left panel shows low‐magnification images. High magnification of immunofluorescent staining in the white box showed the mis‐localization of MyRF with the truncated TDP‐35 indicated by FLAG in the cytoplasm in the right panel (*n* = 3, male mice of 6–10 months old for 2 months injection in the corpus callosum). Scale bar: 0.5 μm (low magnification) and 0.1 μm (high magnification). (B) Western blotting analysis showing an increase of cytoplasmic and decrease of nuclear MyRF in the truncated TDP‐35 expressed mouse brain, as compared with the WT mouse. GAPDH and Histone H3 are cytoplasmic and nuclear marker proteins, respectively. (C) The relative levels of MyRF in the cytosolic (ratios of cytoplasmic MyRF to GAPDH) and the nucleus (ratios of nuclear TDP‐43 to Histone H3) fractions (*n* = 4 per group, male C57BL/6 mice at 6–10 months of age for 1‐month injection on corpus callosum). One‐way ANOVA followed with Tukey's test. MyRF in nucleus **p = 0.0031 (*t* = 4.763, df = 6). MyRF in cytoplasm ***p < 0.001 (*t* = 14.68, df = 6). Data are mean ± SEM. (D) Western blotting analysis showing an increase of cytoplasmic and decrease of nuclear N‐terminal MyRF in the truncated TDP‐35 transfected HEK293 cells, as compared with the WT mouse. Tubulin and Histone H3 are cytoplasmic and nuclear marker proteins, respectively. (E) The relative levels of N‐terminal MyRF in the cytosolic (ratios of cytoplasmic MyRF to Tubulin) and the nucleus (ratios of nuclear TDP‐43 to Histone H3) fractions. The data are obtained from 4 independent experiments. One‐way ANOVA followed with Tukey's test. MyRF in nucleus ***p = 0.0002 (*t* = 8.016, df = 6). MyRF in cytoplasm ***p<0.001 (*t* = 13.59, df = 6). The data are presented as mean ± SEM.

It is known that MyRF can be self‐cleaved in cytoplasm, and then moves into nucleus to activate myelin gene transcription in oligodendrocytes [42, 43, 44, 45]. Thus, we investigated whether the accumulation of pathological TDP‐43 in cytoplasm influenced the distribution of endogenous MyRF. Through the immunofluorescent staining of the expressed TDP‐35 in mouse brain, we found that the mis‐localization of MyRF with the truncated TDP‐35 in the cytoplasm of mouse brain in vivo (Figure 5A). Western blotting also showed the increase of cytoplasmic MyRF, following with the decrease of nuclear MyRF by the truncated TDP‐35 injected mouse brain (Figure 5B, C), which was confirmed by the co‐transfection of N‐terminal MyRF and truncated TDP‐35 in the HEK293 cells (Figure 5D, E). Given that MyRF is a transcription factor specifically expressed in oligodendrocytes to regulate the expression of oligodendrocyte‐specific genes, it is plausible that truncated TDP‐43 in oligodendrocytes could interact with MyRF, thereby impacting its transcriptional regulation of genes involved in oligodendrocyte function, including myelin‐associated genes. This interaction may contribute to demyelination in oligodendrocytes. However, it is important to note that this hypothesis requires further investigation and experimental testing to validate its validity.

## 4 DISCUSSION

The cytoplasmic TDP‐43 accumulation is mainly characterized in the brains and spinal cords of nearly all patients (~97%) with ALS and in ~45% of FTLD cases [1, 28, 46, 47, 48]. In addition, 57% of AD cases and some dementia patients with Lewy bodies also show this cytoplasmic pathology of TDP‐43 in their brains [20, 21, 22, 49, 50]. As the mis‐localization of TDP‐43 in cytoplasm can lead to the nuclear depletion of TDP‐43 in ALS and FTLD [1, 28, 51], as well as other neurological disorders [19, 20, 21, 22, 49], the combination of cytoplasmic inclusions has been thought to critically contribute to TDP‐43's neuropathology [52]. Thus, to better understand and treat these TDP‐43‐associated diseases, research is urgently needed to resolve whether and how the gain of abnormal function is the salient mechanism underlying neurodegeneration [15]. The cytoplasmic accumulation of TDP‐43 appears to be independent of its mutations because only <5% of ALS patients carry mutations in TDP‐43 [10, 19, 53, 54]. Thus, pathological conditions other than TDP‐43 mutations are the major factors responsible for the cytoplasmic inclusions, and that mutations in TDP‐43 may exacerbate this abnormal redistribution. Using non‐human primates, the expression of TDP‐43 in the brain and spinal cords of monkeys mediated by AAV vector led to the obvious cytoplasmic distribution of TDP‐43 [17, 18], which are similar to those seen in patient brains [19, 20, 21, 22]. Therefore, it provided us an ideal model to explore whether and how the cytoplasmic TDP‐43 dysfunction affects neuropathology thoroughly.

Using the TDP‐43 monkey generated previously [18], we demonstrated that the presence of cytoplasmic TDP‐43 fragments under UBC promoter significantly affects axonal guidance pathway in white matter by the high‐throughput transcript profiling. Axons are guided by a variety of factors, such as netrins, ephrins, slits, and semaphorins. These guidance cues are read by growth cone receptors and signal transduction pathways downstream to elicit changes in cytoskeletal organization that determine which way the growth cone will turn. Actually, in terms of TDP‐43 associated diseases, the oligodendrocyte morphological defects can be seen and accompanied by abnormal myelin sheath structure and large number of inclusions in ALS patients [23, 24, 25, 26, 27], and even the damaged myelin structures were found in the differentiated NG2+ precursor cells [23]. Through the pathological detection of intracellular aggregates, TDP‐43 is often positively stained in oligodendrocytes and exhibits characteristics similar to the accumulation of TDP‐43 in cytoplasm of the neuronal cells [28, 29, 30, 31, 32, 33, 34]. Such as in the anterior horn region of the spinal cord of ALS cases [35], the ALS related FOSMN syndrome patients [36], the deep cortical white matter region of FTLD‐TDP brain [25], or even in oligodendrocytes of AD and Lewy body dementia [37]. To better explore the question of whether and how the cytoplasmic accumulation of TDP‐43 in oligodendrocytes could cause pathological demyelination thoroughly, in the current study, we expressed the mutant TDP‐43 in oligodendrocytes specifically and found that, the truncated TDP‐43 fragments contributed to the impairment of axonal integrity through demyelination in white matter of monkey brain. As expected, in the formed cytoplasm inclusions, the truncated TDP‐35 could interact with the functional MyRF, as a consequence, to downregulate the myelin‐associated genes expression at the transcriptional level. It was consistent with the critical role of the C‐terminal glycine‐rich domain of TDP‐43, associated with a large number of downstream binding proteins, to loss their normal physiological functions [9, 10, 12].

Although oligodendrocytes play a critical role in myelination, the orchestration of brain myelination is complex and multifactorial, as this process is regulated by a number of distinct factors such as extracellular growth factors, matrix proteins, and electrical activity [55, 56]. It is known that MyRF can be self‐cleaved on the endoplasmic reticulum (ER) and then translocated from cytoplasm into nucleus to execute the transcription factor role on myelin‐specific genes in oligodendrocytes [42, 43, 44, 45]. Previous reports noted that overexpression of TDP‐43 or other misfolded proteins induces ER stress [57, 58, 59]. As an ER membrane‐bound enzyme and responsible for the cleavage of TDP‐43, Caspase‐4 is also activated under ER stress [60], which can be triggered by protein misfolding, aging, oxidative stress, and many environmental insults [57, 58, 59]. More importantly, in the early reports, caspase‐4 and markers of ER stress are up‐regulated in the spinal cords of patients with sporadic ALS [61]. Although the mechanism behind remains to be investigated, these changes still reflect the toxic effect of accumulation of misfolded proteins in oligodendrocytes. Thus, our results are in agreement with that the truncated TDP‐43 in cytoplasm also interacted with MyRF, to cause a decrease of myelin proteins and subsequent loss of myelin, suggesting that the production of myelin proteins in mature oligodendrocytes is important for the maintenance of myelin in adult brains as well [62].

It was known that the N‐terminal fragment of MYRF (nMYRF) is generated by cleavage of full‐length MYRF in the cytoplasm and then moves into nucleus to activate myelin gene transcription in oligodendrocytes [42, 43]. Similar to mutant HTT that can bind nMYRF to prevent its transcriptional activity in the nucleus [41, 63], cytoplasmic TDP‐43 is likely to interact with nMyRF to reduce its nuclear distribution and function. However, the binding sites for the TDP‐43 and nMyRF interaction remain to be defined.

Although our findings suggest that cytoplasmic TDP‐43 can influence the myelination function of oligodendrocytes, it is important to note that nuclear TDP‐43 also disrupts the expression of various genes, which can directly or indirectly contribute to myelination defects in oligodendrocytes [64, 65, 66]. Therefore, further investigations are necessary to elucidate the intricate effects of nuclear TDP‐43 and to determine how both nuclear and cytoplasmic TDP‐43 specifically impact the function of oligodendrocytes and lead to impaired myelination.

## AUTHOR CONTRIBUTIONS

PY and X‐JL designed research. LZ, DB, XW, KO, and QJ performed research and analyzed data. BL performed monkey's surgeries. ZT and JL assisted on the monkey's MRI and analysis. DH, SY, LW, and SL provided technical guidance. PY and X‐JL wrote the paper.

## CONFLICT OF INTEREST STATEMENT

The authors declare that they have no conflict of interest.

## CONSENT FOR PUBLICATION

All authors have approved the content of this manuscript and provided consent for publication.

## Supporting information


**FIGURE S1.** The general expressing mutant TDP‐43 affects axon guidance in monkey brain. (A) Volcanic map analysis of the differentially expressed genes (DEGs) from the high‐throughput transcriptome profiling of the general expression of TDP‐43(M337V) and GFP in the injected white matter of monkey brain under the UBC promoter. Colored points indicate the significant DEGs statistically [false discovery rate (FDR) <0.05]. Green dots are the down‐regulated genes [log2(fold change) <−0.8], red dots are the up‐regulated genes [log2(fold change) >0.8], blue dots are the unaltered genes [−0.8<log2(fold change) <0.8]. *n* = 3 biological replicates per genotype. The cortical TDP‐43 overexpression in monkey had 1559 up‐regulated genes, 1523 down‐regulated genes, and 22,327 unchanged genes as compared with the GFP controls (male Cynomolgus macaques of 8–12 years old for 2 months injection in the corpus callosum). (B) The enriched DEGs with “Axon guidance” by KEGG pathway were clustered after TDP‐43(M337V) expressing in the injected white matter of monkey when compared with GFP control. The red font highlight indicated the BgRatio and Count on the “Axon guidance”‐associated cluster. (C) The DEGs in TDP‐43(M337V) generally express white matter of the injected monkey brain. Heatmap showed the DEGs, related to the “Axon guidance” or “Amyotrophic lateral sclerosis (ALS)”, in the TDP‐43(M337V) expressing white matter of monkey brain when compared with GFP control.
**Figure S2.** The statistical graph of the ROI of the monkey's brain. It was shown that the ROI distribution of the minimum, maximum, 10th, 90th, median, skewness (skew), and kurtosis values were calculated for the AAV‐MBP‐GFP and AAV‐MBP‐TDP‐43 injected monkey's brain (*n* = 3, male Cynomolgus macaques of 8–12 years old for 2 months injection in the corpus callosum).
**Figure S3.** The specific expression of mutant TDP‐43 in oligodendrocytes of monkey brain. (A) The double immunofluorescent staining of the monkey corpus callosum injected with AAV‐MBP‐TDP‐43, using the antibodies to TDP‐43 (green) with Oligo2 (red). The nuclei were stained with DAPI (blue). Representative images were obtained from the male 8–12‐years‐old monkeys. Note that the co‐localization of TDP‐43 (arrow pointed) with the oligodendrocyte marker (Oligo2) showed the successfully and specifically expression of TDP‐43(M337V) in the oligodendrocytes of monkey brain. Scale bar: 10 μm. (B) Western blotting of anti‐FLAG and anti‐TDP‐43 shows that the expressing TDP‐43(M337V) promoted the cleavage of TDP‐35 fragments, and led to the significant reduction of the myelin‐associated proteins of MBP and PLP1, but no alter the expression of the total MyRF and Oligo2. Tubulin and Vinculin served as a loading control (male Cynomolgus macaques of 8–12 years old for 2 months injection in the corpus callosum).
**Figure S4.** The expression of the truncated TDP‐43 was C‐terminal, but not N‐terminal fragments. (A) The TDP‐35 fragments transfected in HEK293 were not determined by the N‐terminal TDP43 antibody (E2G6G, 89718S and E‐10, sc‐376311), but could be proved by the C‐terminal TDP‐43 antibody (12892‐1‐AP and G400, 3448S). (B) The TDP‐35 fragments injected in mouse brain were not determined by the N‐terminal TDP43 antibody (E2G6G, 89718S and E‐10, sc‐376311), but could be re‐proved by the C‐terminal TDP‐43 antibody (12892‐1‐AP and G400, 3448S).
**Figure S5.** The specific expression of mutant TDP‐35 in oligodendrocytes of mouse brain. (A) The double immunofluorescent staining of the monkey corpus callosum injected with AAV‐MBP‐TDP‐35, using the antibodies to TDP‐43 (green) with Oligo2 (red). The nuclei were stained with DAPI (blue). Representative images were obtained from the male mice at 6–10 months of age for 1 month injection in the corpus callosum. Note that the co‐localization of TDP‐43 (arrow pointed) with the oligodendrocyte marker (Oligo2) showed the successfully and specifically expression of TDP‐35 (M337V) in the oligodendrocytes of mouse brain. Scale bar: 10 μm. (B) Western blotting of anti‐FLAG and anti‐TDP‐43 shows that the expressing TDP‐35 fragments, led to the significant reduction of the myelin‐associated proteins of MBP and PLP1, but no alter the expression of the total MyRF and Oligo2. Tubulin and Vinculin served as a loading control (male mice of 6–10 months of age for 1 month injection in the corpus callosum).
**Figure S6.** Truncated TDP‐35 in oligodendrocyte leads to demyelination in mouse brain. (A) Electron microscopy revealed the obvious demyelination or axonal degeneration in mice brain injected with AAV‐MBP‐TDP‐35, as compared with AAV‐MBP‐GFP. Scale bar: 0.5 μm (low magnification) and 0.1 μm (high magnification) (male mice of 6–10 months old for 2 months injection in the corpus callosum). (B) The *g*‐ratio was deteriorated in the TDP‐35 expressed mouse brain (*g* = 0.0066 ± 0.8235), as compared to the GFP control (*g* = 0.0109 ± 0.6791). At least 100 axons per genotype were examined.

## Data Availability

The key data supporting the findings of this study are presented within the article and the supplemental materials.
