# Supplementary figures and images for: Pathologic TDP‐43 downregulates myelin gene expression in the monkey brain
Source: Brain Pathol. 2024 May 23;34(6):e13277. doi: 10.1111/bpa.13277 (PMC11483520; doi:10.1111/bpa.13277)

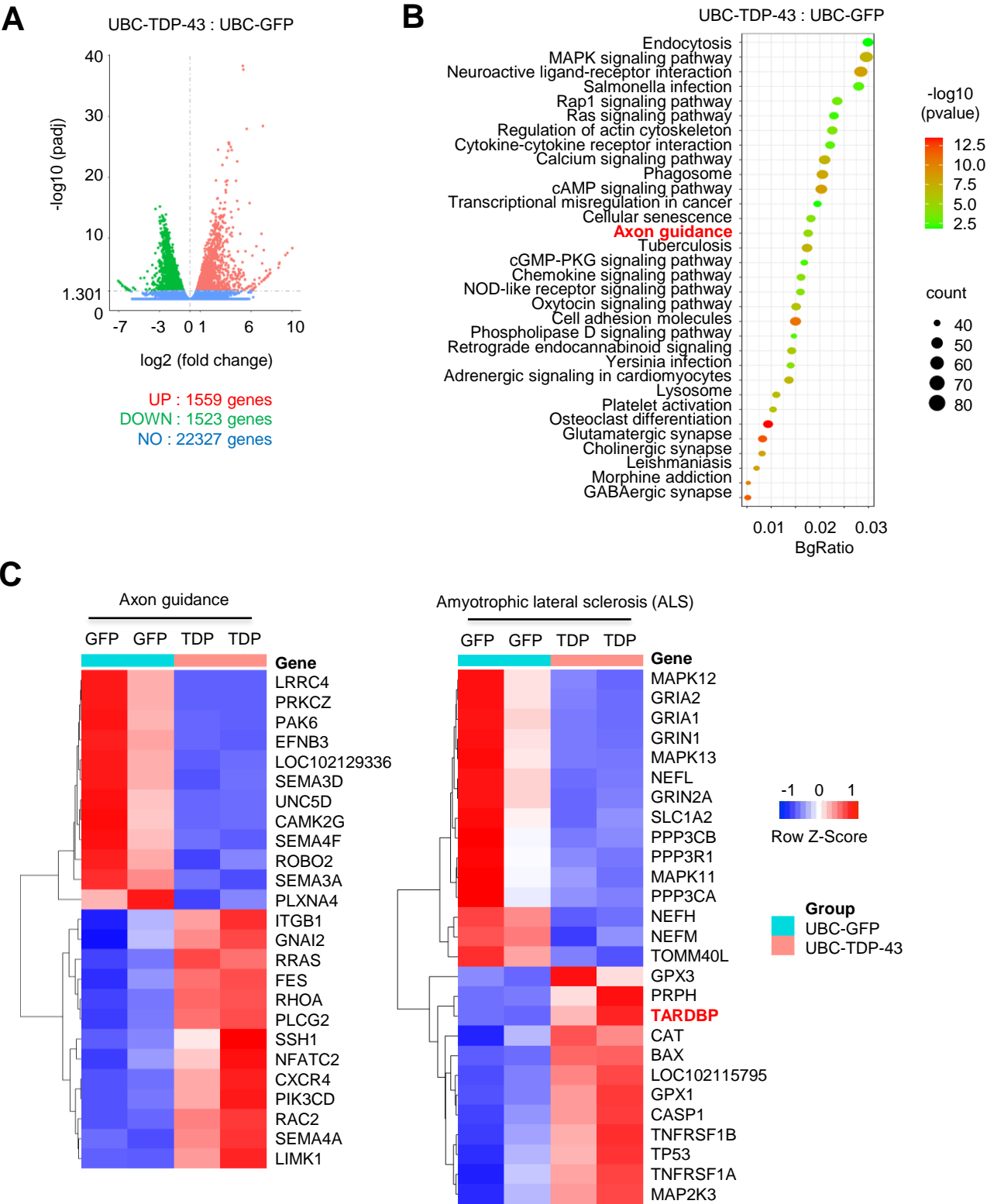

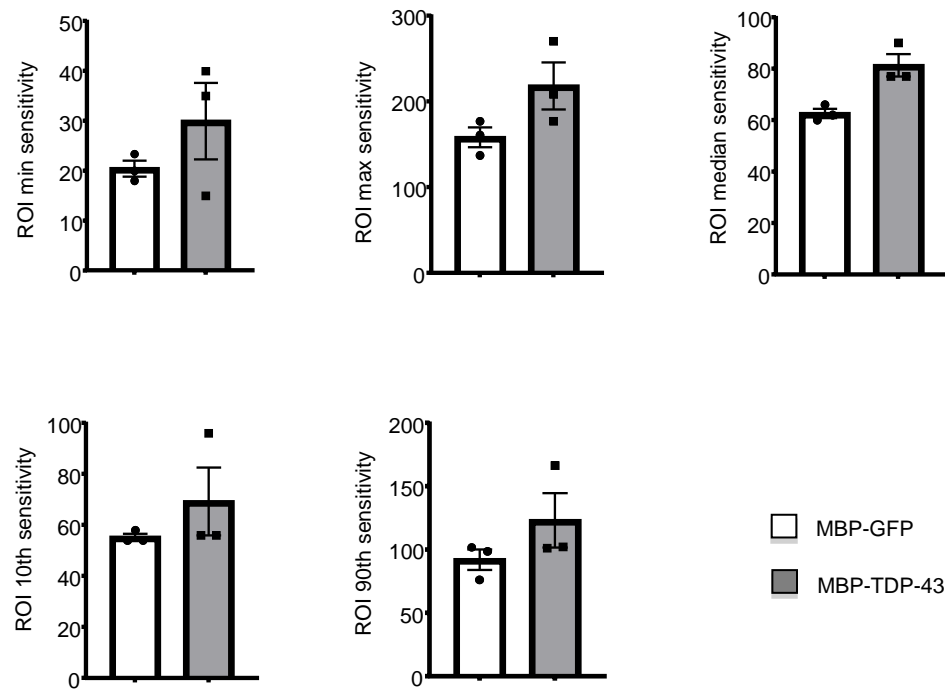

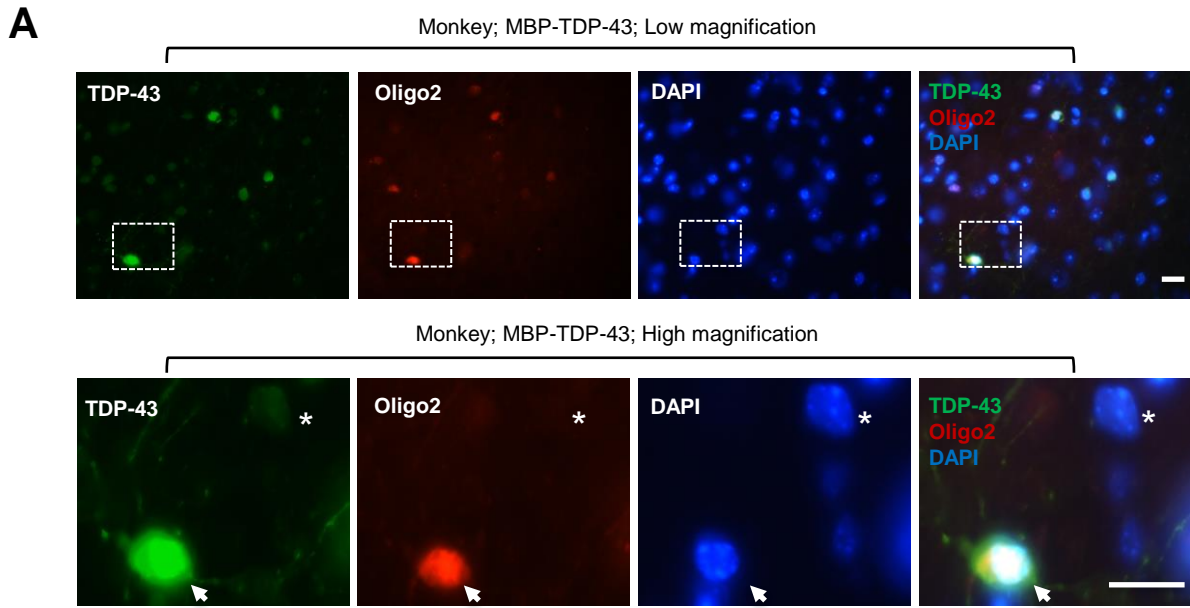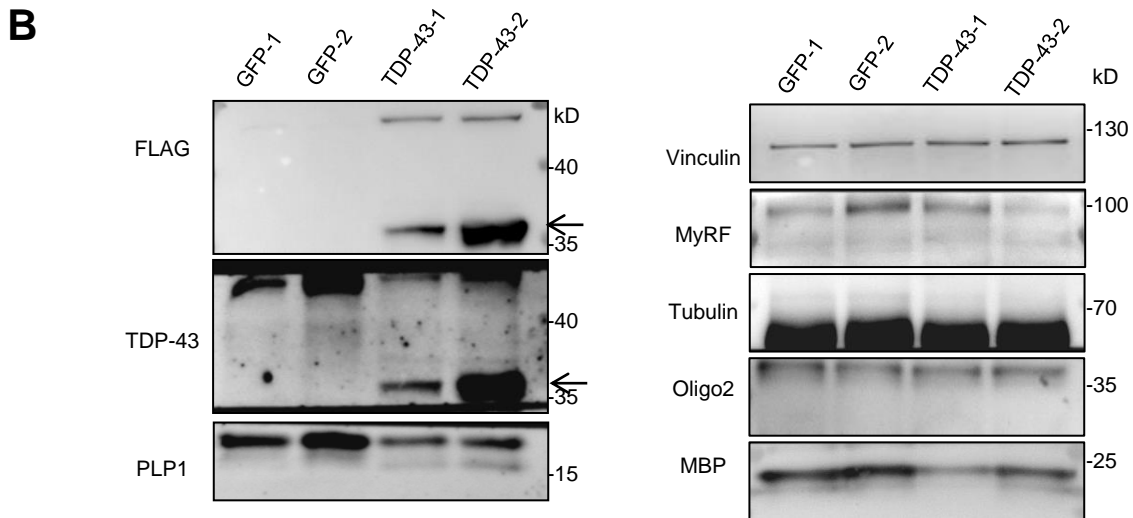

**A**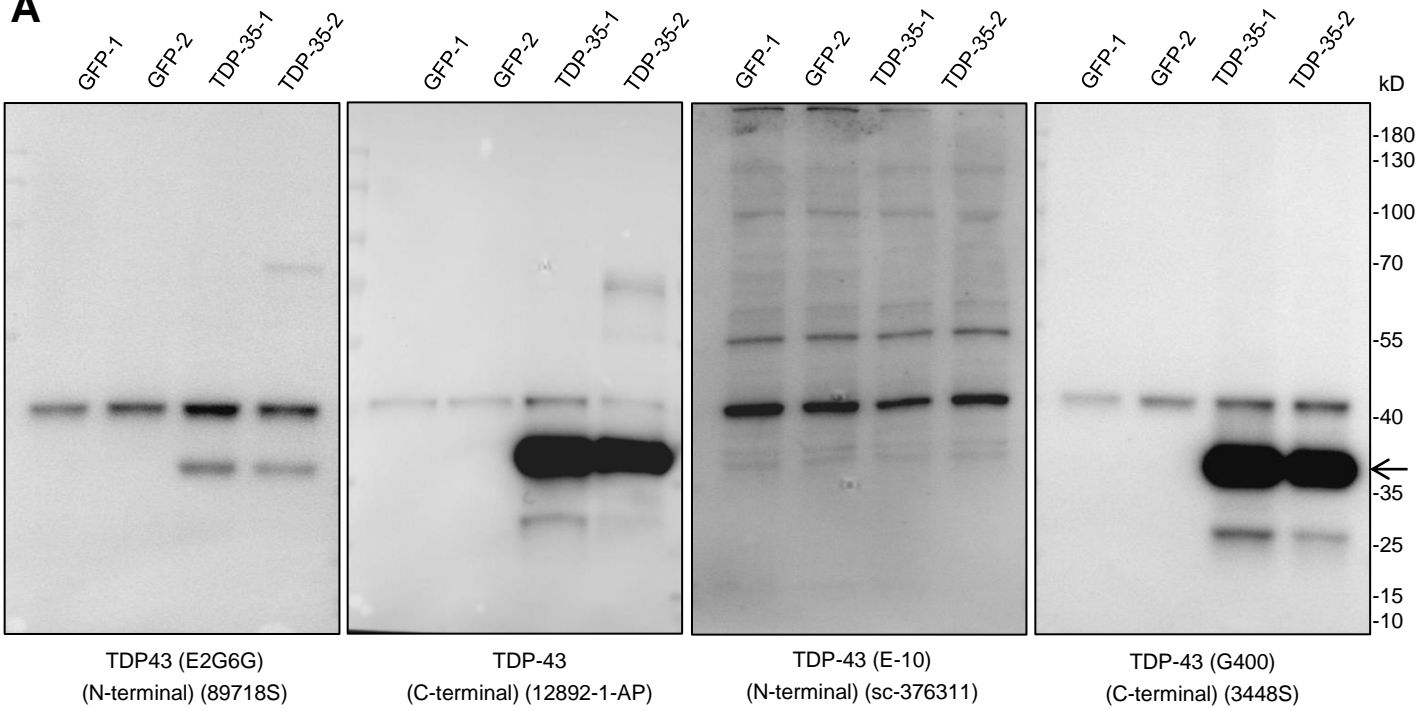**B**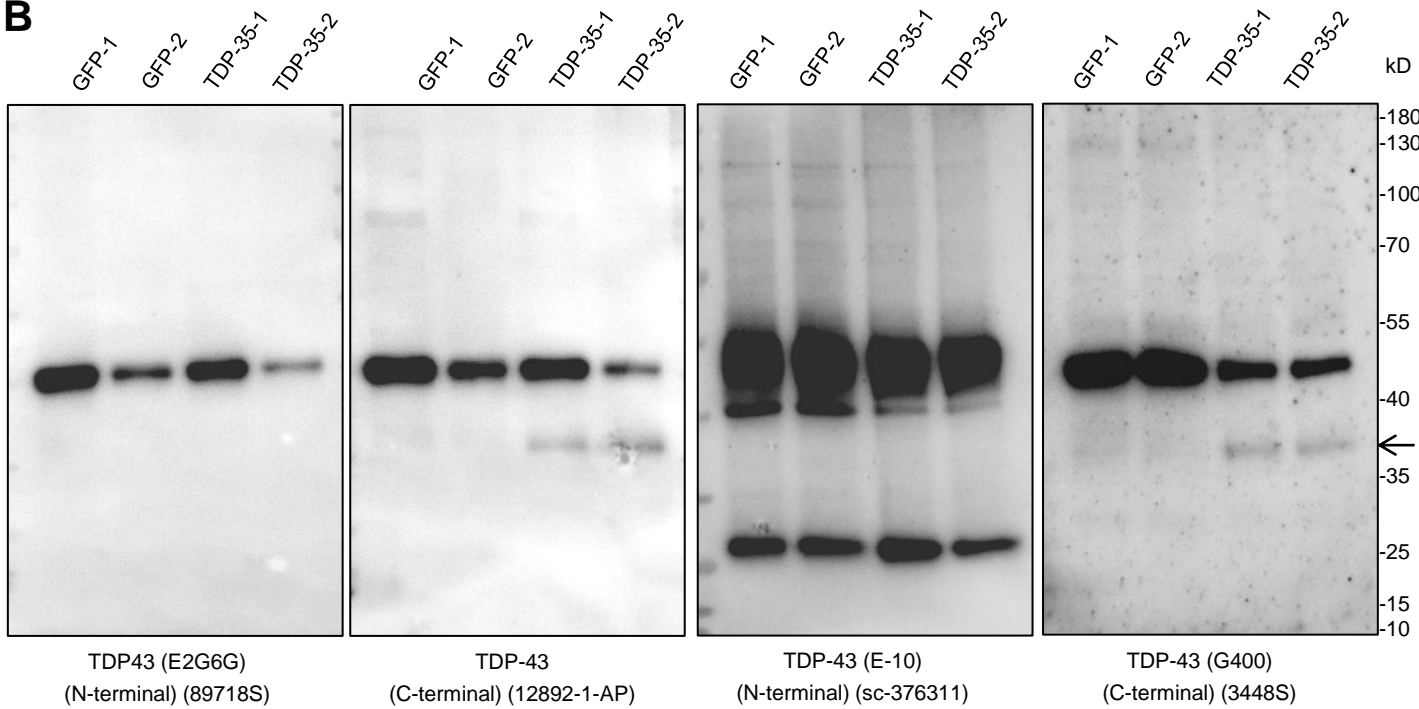

**A**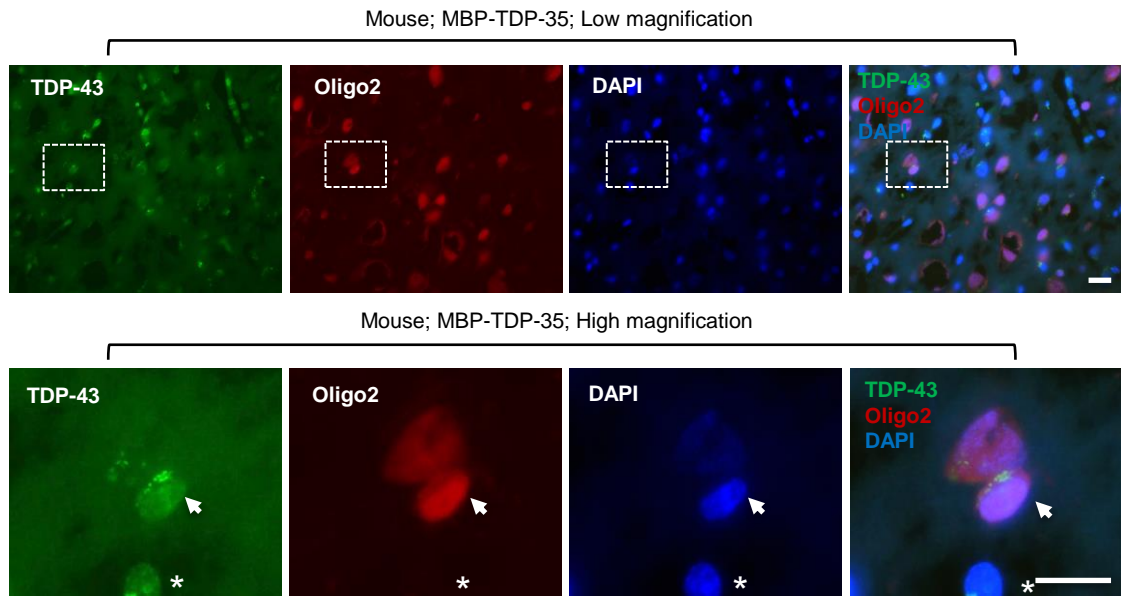**B**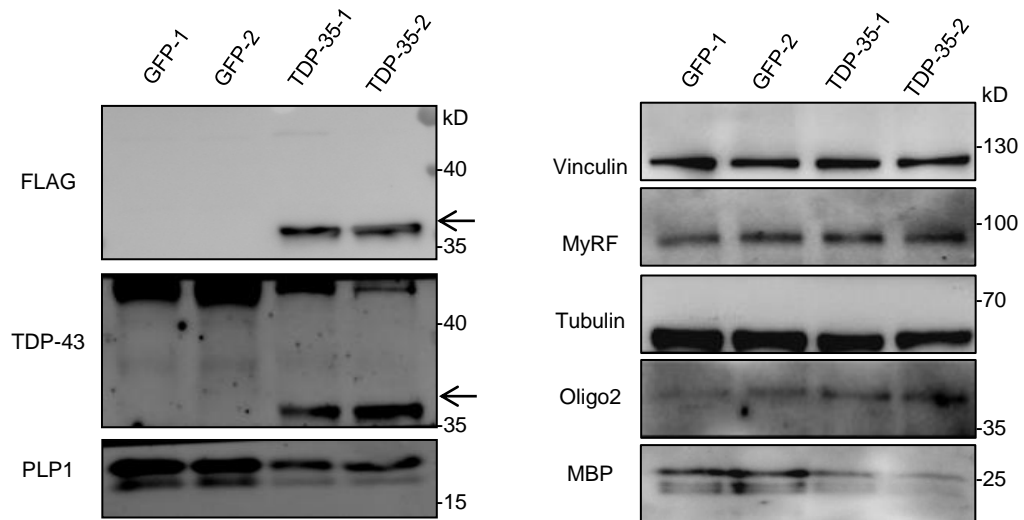

**A**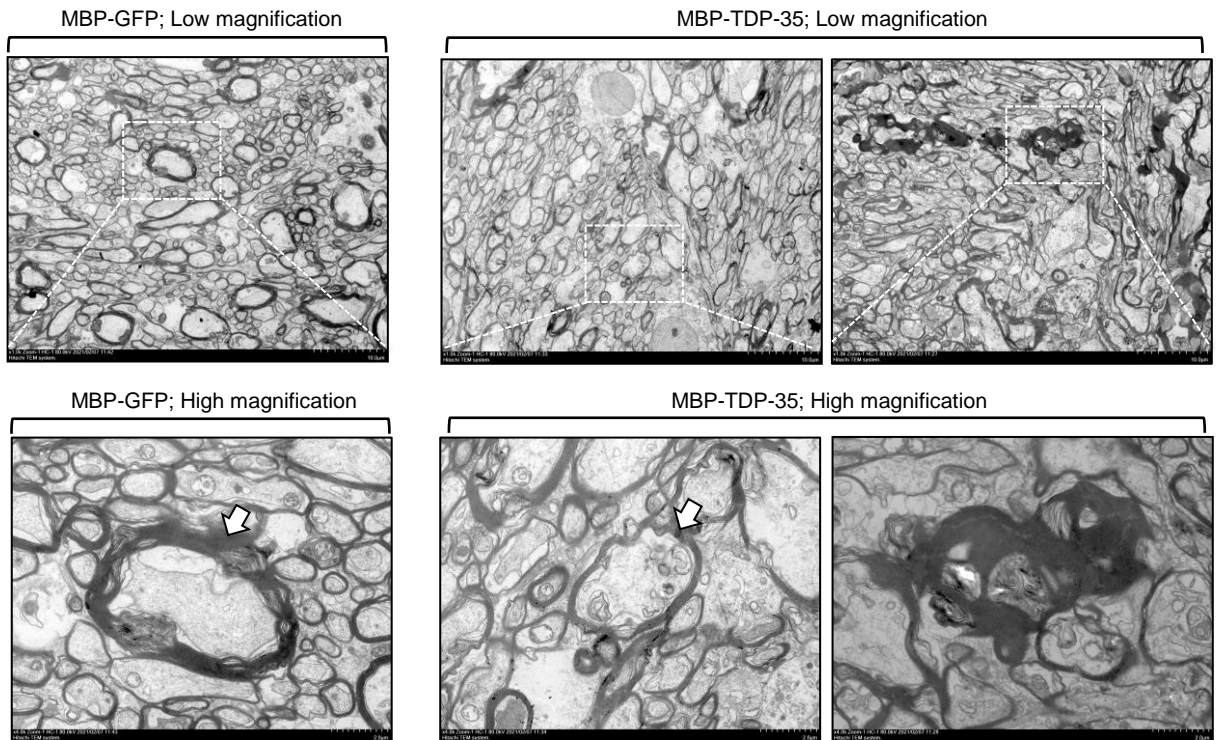**B**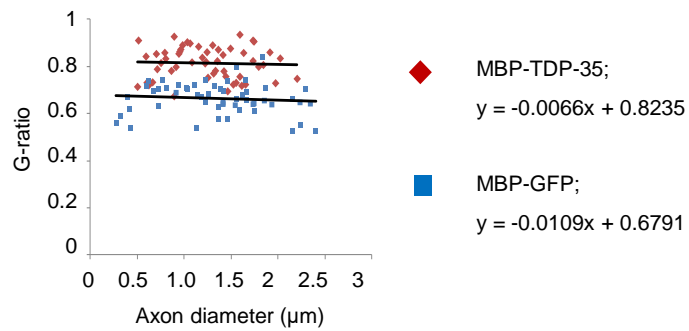

Supplement: Supplementary file 1 — FIGURE S1. The general expressing mutant TDP‐43 affects axon guidance in monkey brain. (A) Volcanic map analysis of the differentially expressed genes (DEGs) from the high‐throughput transcriptome profiling of the general expression of TDP‐43(M337V) and GFP in the injected white matter of monkey brain under the UBC promoter. Colored points indicate the significant DEGs statistically [false discovery rate (FDR) <0.05]. Green dots are the down‐regulated genes [log2(fold change) <−0.8], red dots are the up‐regulated genes [log2(fold change) >0.8], blue dots are the unaltered genes [−0.8<log2(fold change) <0.8]. n = 3 biological replicates per genotype. The cortical TDP‐43 overexpression in monkey had 1559 up‐regulated genes, 1523 down‐regulated genes, and 22,327 unchanged genes as compared with the GFP controls (male Cynomolgus macaques of 8–12 years old for 2 months injection in the corpus callosum). (B) The enriched DEGs with “Axon guidance” by KEGG pathway were clustered after TDP‐43(M337V) expressing in the injected white matter of monkey when compared with GFP control. The red font highlight indicated the BgRatio and Count on the “Axon guidance”‐associated cluster. (C) The DEGs in TDP‐43(M337V) generally express white matter of the injected monkey brain. Heatmap showed the DEGs, related to the “Axon guidance” or “Amyotrophic lateral sclerosis (ALS)”, in the TDP‐43(M337V) expressing white matter of monkey brain when compared with GFP control. Figure S2. The statistical graph of the ROI of the monkey's brain. It was shown that the ROI distribution of the minimum, maximum, 10th, 90th, median, skewness (skew), and kurtosis values were calculated for the AAV‐MBP‐GFP and AAV‐MBP‐TDP‐43 injected monkey's brain (n = 3, male Cynomolgus macaques of 8–12 years old for 2 months injection in the corpus callosum). Figure S3. The specific expression of mutant TDP‐43 in oligodendrocytes of monkey brain. (A) The double immunofluorescent staining of the monkey corpus callosum injec [file BPA-34-e13277-s001.pdf]
